# Disabilities in leprosy: an open, retrospective analyses of institutional records^☆^^☆☆^

**DOI:** 10.1016/j.abd.2019.07.001

**Published:** 2019-12-28

**Authors:** Santoshdev P. Rathod, Ashish Jagati, Pooja Chowdhary

**Affiliations:** a Department of Dermatology, Smt. NHL Municipal Medical College, V. S. Hospital, Ahmedabad, Gujarat, India; b Consultant Dermatologist, Twachha Skin Clinic, Ahmedabad, Gujarat, India

**Keywords:** Leprosy, Social stigma, Statistics on sequelae and disability

## Abstract

**Background and objectives:**

Leprosy remains a leading cause of peripheral neuropathy and disability in the world. Primary objective of the study was to determine the incidence of deformities present at a time of diagnosis and new deformities that patients develop over follow up period.

**Material and methods:**

An open, retrospective cohort study was performed at a tertiary medical center in western India. Recruitment phase of the study was of 2 years (2009–2010) followed by observation/follow up phase of 7 years till 31st December 2017. New patients with leprosy and released from treatment cases who presented with deformity as defined by WHO disability grade (1998) and subsequently developing new deformities during the follow up period of up to 7 years were included in the study.

**Results:**

The study included 200 leprosy patients. Of the total 254 deformities, 168 (66.14%) deformities were noticed at the moment of diagnosis, 20 (7.87%) deformities occurred during the follow up phase. Of all patients, 21.25% had Grade 1 deformity and 6.31% had Grade 2 or more severe deformity. Deformities of hand were most common in 44.48%, followed by feet 39.76%, and face 15.74% respectively.

**Limitation of study:**

Mode of inclusion of patient was self-reporting during follow up phase so there is possible under reporting of the disabilities.

**Conclusion:**

New deformities continue to develop in certain forms of leprosy even after release from treatment. Long-term & regular follow up of patients who have been released from treatment is required.

## Introduction

Among communicable diseases, leprosy remains a leading cause of peripheral neuropathy and disability in the world, despite extensive efforts to reduce the disease burden. Widespread implementation of MDT has clearly been extremely successful in curing and reducing the prevalence of leprosy throughout the world including India.^1^ As on 1st April’17 the prevalence rate of leprosy in India was 0.66/10,000. The registered global prevalence of leprosy globally was 192,713 cases (0.25/10,000 population) at the end of 2017, an increase by 20,765 cases over that in 2016.^2^ In recent years under leprosy control program more attention has been given to prevention of disability. Current focus of health authorities and governments across the globe is on tackling stigma due to Leprosy. One of the major factors responsible for stigmatization of leprosy patients is their identification in society due to visible deformities.

Disability is a broad term covering any impairment, activity limitation or participation restriction affecting a person. Physical disability in leprosy is defined by the WHO in three categories, WHO grading system^3^: Grade 0 – absence of disability (no anesthesia) and no visible damage or deformities on eyes, hands and feet; Grade 1 – loss of protective sensibility in the eyes, hands or feet, but no visible damage or deformities; and Grade 2 – presence of deformities or visible damage to the eyes (lagophthalmos and/or ectropion, trichiasis, corneal opacity, visual acuity less than 0.1 or difficulty counting fingers at 6 meters), visible damage on hands or feet (hand with ulcerations and/or traumatic, resorption, claw, fallen hand, ulcers; feet with trophic and/or traumatic injuries, resorption, claw, foot drop, ulcers, ankle contracture).^4^

Disability assessment is a very relevant measure of leprosy control. However, routinely in our healthcare System only G2D record (WHO) is maintained, whereas Grade 1 assessment though is more important in terms of prevention of disability, is a neglected issue. Therefore timely diagnosis of Grade 1 disability is urgently required for disability limitation and mitigation. Searching for associated factors will definitely help to lessen the suffering of many leprosy patients. With this perspective current cross-sectional study was carried out.

## Materials and methods

This was an open, retrospective cohort study performed at a tertiary medical center in western India.

### Study population

Comprised of patients presenting with Hansen's disease having disabilities and deformities at the time of diagnosis and those subsequently developing new disabilities during the follow up phase of 7 years.

### Inclusion criteria

Patients presenting with disability and deformities as defined by World Health Organization (WHO) were included in the study. Recruitment phase of the study was of 2 years (2009–2010) followed by observation/follow up phase of 7 years till 31st December 2017. Patients subsequently developing new deformities during the follow up period of up to 7 years were included in the study. During the follow up phase, patients who had completed anti-leprosy treatment during the recruitment phase were further followed up for 7 years. No other patients were added to the study cohort. The mode of inclusion was self-reporting by patients followed by clinical assessment for deformity. In the present study, outcome was physical disability expressed by different indicators such as number of new cases detected with occurrence of Grade 1 and Grade 2 deformity. Assessment for presence and severity of deformity was done at time of presentation for diagnosis and treatment of leprosy and during the follow up visits.

### Exclusion criteria

Traumatic deformities due to road traffic accidents in patients with leprosy were excluded from the study. The study was conducted as a part of thesis protocol submitted to the university. Patient consent from all study participants were taken for use of medical records and clinical photographs when necessary.

## Results

Of the 200 patients followed up in the study, 134 (67%) were males and 66 (33%) female. Lepromatous Leprosy was the most common type of leprosy in the current study with 61 (30.5%) followed by Tuberculoid Leprosy 54 (27%), borderline lepromatous leprosy 42 (21%), borderline tuberculoid leprosy 29 (14.5%), pure neuritic leprosy 6 (3%) respectively and mid-borderline leprosy 1 (0.5%), indeterminate leprosy 1 (0.5%) each. Proportion of Multibacillary (MB) cases was 67% as compared to 33% Paucibacillary (PB) cases.

Nerve involvement was assessed at baseline and at each visit thereafter. Assessment of nerve function was done by sensory examination with Semmes Weinstein (SW) Monofilaments, nerve palpation and assessment of motor function by Medical Research Council (MRC) grading system. Impairment in the nerve function is the chief reason for development of disability and that's why it is important to know extent, type and site of nerve involvement. Ulnar nerve in the upper limb was the most commonly involved nerve in overall 83.5% of total patient in the study, involvement was symmetrical in 121 (60.5%) and asymmetrical in 46 (23%) patients followed by involvement of Anterior Tibial nerve in 152 (76%) of patients, Lateral popliteal nerve in 106 (53%) patients, great auricular nerve in 34 (17%) and supra-clavicular nerve in 7 (3.5%) of patients respectively (Table 1).

**Table 1:** Nerve involvement.

Nerve	Symmetrical	Asymmetrical
Ulnar	121 (60.5%)	46 (23%)
Anterior tibial nerve	92 (60.52%)	60 (39.48%)
Lateral popliteal	70 (35%)	36 (18%)
Greater auricular	26 (13%)	8 (4%)
Supra-clavicular	6 (3%)	1 (0.5%)
Others	4 (2%)	5 (2.5%)
Supra-orbital	2 (1%)	2 (1%)

Upon disability assessment by WHO grading system, 152 (76%) had no visible impairment, 21.25% of the patients had Grade 1 or above deformities and 16 (6.31%) had Grade 2 or more severe deformity. Of these deformities, deformities involving hand were 113 (44.48%), feet were 101 (39.76%) and involving face were 40 (15.74%) respectively. This suggests hand and feet are more commonly affected by deformities due to repetitive trauma due to work and house hold deeds (Table 2).

**Table 2:** Grade of deformities in current study.

Grade	% of total functional deformities	Number of patients affected with deformities (of total 200 study participants)
Grade 1 deformity	22	17% (*n* = 34)
Hand	16	
Feet	16	
Face	21.25% (*n* = 54)	

Grade 2 deformity	08	07% (*n* = 14)
Hand	04	
Feet	04	
Face	6.31% (*n* = 16)	

Total	27.56% (*n* = 70) of all disabilities	

Deformities in Hansen's disease can be further sub-divided in the functional deformity and anatomical/morphological deformity. Most common functional deformity was trophic ulcer of hand or foot seen in 54 (29.34%) patients followed by claw hand 21 (11.41%), foot drop 2 (1.1%) and wrist drop 1 (0.54%). Amongst the morphological deformities, leonine facies 14 (7.60%), Ear deformity 13 (7.06%), resorption of digits 6 (3.20%), madarosis 6 (3.20%), sagging face 4 (2.17%), saddle nose 3 (1.63%), eye deformities 3 (1.63%) (Table 3).

**Table 3:** Functional & anatomical/morphological deformities in the current study.

*Functional deformities*	
Trophic ulcers	18
Upper limb	32
Lower limb	17
Claw hand	01
Wrist drop	02
Foot drop	18
Total	70

*Anatomical/morphological deformities*	
Leonine facies	14
Sagging face	04
Saddle nose	03
Madarosis	06
Eye deformities	03
Mega-globules of ear	13
Resorption of digits	06
Total	46

Of the 254 disabilities, 168 (66.14%) deformities were noticed at the moment of diagnosis, 20 (7.87%) deformities occurred while patient was on anti-leprosy treatment with Multidrug Therapy (MDT) for 1 year and (21.25%) deformities occurred in patients who were released from treatment and were diagnosed during the follow up period.

## Discussion

After achieving national elimination target, the focus of leprosy care is now shifting from simply providing anti-leprosy treatment to the affected patients to dealing with consequences of leprosy and especially prevention of disability due to leprosy.^5^ The findings in the current study showed that proportion of leprosy patients with disability was quite high, 21.54% had Grade 1 and 6.31% had Grade 2. Previous studies by Sarkar et al.^5^ in India and Schreuder et al.^6^ in Thailand showed similar prevalence of Grade 1 and Grade 2 disability. At National level to assess program effectiveness only Grade 2 disability record is maintained but for prevention of disability, Grade 1 assessment is more important. Because, before visible deformity (Grade 2) occurs, nerve function impairment definitely occurs (sensory, motor or both) i.e. those patients with G2D must have passed through the stage of Grade 1. Therefore while examining any leprosy case, after examination of skin lesions; thorough neurological examination of peripheral nerves is essential. According to global leprosy strategy 2016–2020, the following are the targets envisaged by the Strategy by 2020: 1) Zero Grade 2 Deformity (G2D) among pediatric leprosy patients; 2) Reduction of new leprosy cases with G2D to less than one case per million population; 3) Zero countries with legislation allowing discrimination on basis of leprosy.^7^ Visible deformities and disabilities have been found to be the prominent contributor of stigma development in leprosy affected persons^8^ while it also triggers the development of negative attitudes toward leprosy among unaffected people.^9^ The number of cases with G2D at the time of diagnosis directly reflects delay in case detection and lack of awareness of leprosy in the community, the capacity of the health system to recognize leprosy early and to some extent the reach of services. One of the targets of the Global Leprosy Strategy is 0 new child cases with G2D. This indicator was reported by 120 countries, of which 88 reported 0 cases, and 27 reported 1–10. Five countries reported more cases: Ethiopia, Mozambique, the Democratic Republic of the Congo, Indonesia and Brazil.^2^

The study reveals higher proportion of MB cases (67%) as compared to PB cases (33%). In recent years such increasing MB ratio trend has been seen in India^10^ and elsewhere. The reasons for this increasing tread of MB ratio could be due to recent aggressive case finding campaigns in countries with high burden of leprosy.^11^ Higher proportion of MB leprosy is in itself a risk factor for development of disability as suggested by studies from by Schreuder (PB – 11%, MB – 33%),^6^ De Oliviera and others (PB – 12%, MB – 37%),^12^ Richardus (PB – 9.8%, MB – 37.6%).^13^

Physical disabilities still remain frequent, however, both at diagnosis and after treatment. Other considerations are necessary to both prevent disabilities and intervene in the evolution of existing disabilities. In our study (66.14%) of deformity were present at time of diagnosis such high proportion of deformity in new cases this reflects delay in the diagnosis and could be seen as a measure of effectiveness of public health program as early detection of leprosy through various information and education activities can prevent deformities via implementation of therapy. Early diagnosis and treatment of the disease and especially reactions remain the main means of preventing the development of physical disabilities.^14^ Multibacillary Leprosy and Impairment at diagnosis was the main risk factor for neurological worsening after treatment/MDT. Early diagnosis and prompt treatment of reactional episodes remain the main means of preventing physical disabilities.

In our study trophic ulcer of hand or foot seen in 29.34% was the most common morphological/anatomical form of deformity. This and incidence of other deformities are similar to study by Nayak et al.^15^ who reported the most common deformity to be trophic ulcer in (21.73%) followed by claw hand, foot drop, madarosis, claw toes, lagophthalmos, ear lobe deformity, facial palsy, and finally nose deformity.

In leprosy, eyes, hands and feet are the commonly affected areas of impairment even in advanced stages. For mobility and other vital activities of daily living, a person has to depend on eyes, hands and feet. In our study, deformities involving hand were 44.48% (*n* = 113), feet were 39.76% (*n* = 101). These findings emphasize the importance of routine assessment of Nerve Function Impairment (NFI) of all new leprosy patients to search for Grade 1 disability. After diagnosis if we can properly educate these patients for self-care like not to walk bare foot, daily inspection of hands/feet for any blisters, red spots, oliohydrotherapy, physiotherapy, eye care, change in occupation, etc. any visible deformity will not occur.

In our study, (21.25%) deformities occurred in patients who were released from treatment and were diagnosed during the follow up period this is primarily due to ongoing nerve damage even after completion of chemotherapy for leprosy. There is an urgent need to develop clinical and serological indicators suggestive of nerve damage and to determine the therapy end-points. Nerve damage is associated with secondary disability.^16^, ^17^ The Duration of Multidrug Therapy (MDT) treatment of leprosy is considered the sole criterion for the cure of the disease, assuming that 12 months of MDT will be sufficient to induce a bacteriological cure.^18^ However, the neurological damage that leads to disabilities and deformities also occurs after therapy is complete. The impairment status worsened in 21.25% of all patients in the 10 years after release from treatment. In a similar study by A.M. Sales et al., showed at the end of the observation period, 40% of patients showed worsening of physical disability after discharge from therapy.^19^ This worsening was associated with the disability grade and the number of skin lesions at diagnosis and with the presence of neuritis. Thus, the worsening of leprosy impairment after specific treatment is principally related to late diagnosis, the extent and severity of the disease and the presence of neuritis. Leprosy-evoked disabilities are more at risk of misdiagnosis and may present to different specialties like plastic surgeons, rheumatologists and vascular surgeons and it usually take a long time for a correct diagnosis and develop complications due to late diagnosis of disease.^20^

In our analysis, where people presented with two or more damaged nerve trunks, secondary disability was more prevalent. During clinical assessment at leprosy diagnosis, the nerves should be palpated, because the number of affected nerve trunks can be used as a proxy indicator of present and future disabilities. This clinical assessment, however, requires skilled health professionals who are familiar with the clinical signs of leprosy.

## Conclusion

New deformities continue to develop in certain forms of leprosy despite therapy. A deformity plays a major role in stigma associated with leprosy. Long-term & regular follow up of patients who have been released from treatment is required. Treatment of leprosy case doesn’t end with completion of multi-drug chemotherapy. The evaluation of the Grade of Physical Disability (GPD) is a practice recommended by the ministry of health and should be performed in all diagnosed patients.^21^ From the clinical point of view, the assessment allows analyzing the impact of the disease in the individual's life, monitoring treatment evolution and establishing strategies for prevention, rehabilitation and self-care. In the epidemiological aspect, it allows to monitor the extent of the endemics, the hidden prevalence and the maintenance of the transmission chain, also reflecting the quality of health care services.^22^

## Financial support

None declared.

## Authors’ contribution

Santoshdev P. Rathod: Statistical analysis; approval of the final version of the manuscript; conception and planning of the study; elaboration and writing of the manuscript; collection, analysis, and interpretation of data; intellectual participation in the propaedeutic and/or therapeutic conduct of the studied cased; critical review of the literature.

Ashish Jagati: Approval of the final version of the manuscript; conception and planning of the study; elaboration and writing of the manuscript; effective participation in research orientation; critical review of the literature; critical review of the manuscript.

Pooja Chowdhary: Statistical analysis; approval of the final version of the manuscript; elaboration and writing of the manuscript; collection, analysis, and interpretation of data; intellectual participation in the propaedeutic and/or therapeutic conduct of the studied cased; critical review of the literature.

## Conflicts of interest

None declared.
